# Efficient Self-Supervised Metric Information Retrieval: A Bibliography Based Method Applied to COVID Literature

**DOI:** 10.3390/s21196430

**Published:** 2021-09-26

**Authors:** Gianluca Moro, Lorenzo Valgimigli

**Affiliations:** Department of Computer Science and Engineering (DISI), University of Bologna, Via dell’Università 50, I-47521 Cesena, Italy; lorenzo.valgimigli@unibo.it

**Keywords:** information retrieval, language model, self-supervised learning, metric learning, healthcare, COVID-19, NLP

## Abstract

The literature on coronaviruses counts more than 300,000 publications. Finding relevant papers concerning arbitrary queries is essential to discovery helpful knowledge. Current best information retrieval (IR) use deep learning approaches and need supervised training sets with labeled data, namely to know a priori the queries and their corresponding relevant papers. Creating such labeled datasets is time-expensive and requires prominent experts’ efforts, resources insufficiently available under a pandemic time pressure. We present a new self-supervised solution, called SUBLIMER, that does not require labels to learn to search on corpora of scientific papers for most relevant against arbitrary queries. SUBLIMER is a novel efficient IR engine trained on the unsupervised COVID-19 Open Research Dataset (CORD19), using deep metric learning. The core point of our self-supervised approach is that it uses no labels, but exploits the bibliography citations from papers to create a latent space where their spatial proximity is a metric of semantic similarity; for this reason, it can also be applied to other domains of papers corpora. SUBLIMER, despite is self-supervised, outperforms the Precision@5 (P@5) and Bpref of the state-of-the-art competitors on CORD19, which, differently from our approach, require both labeled datasets and a number of trainable parameters that is an order of magnitude higher than our.

## 1. Introduction

### 1.1. Overview of the Research

The current COVID-19 pandemic is challenging humanity and we need fast and efficient solutions to handle critical situations. The scientific community constantly needs up-to-date information from a dynamic and growing literature, while the systematic analysis require time and unfeasible efforts of trained professionals in the relevant application domain. In the rapid evolution of events like the ongoing COVID-19 pandemic, extracting and collecting high quality information on items of interest becomes fundamental, but it is complex, even more when data are unlabeled.

Information retrieval systems play a central role in this situation because they can find semantically related documents in a vast collection against a human query. Such systems are built leveraging neural models, but training these models is trivial because they require a collection of papers pre-classified as relevant for a given set of queries or topics. For this reason, labeled datasets, where the relationships between documents and topics are previously known, are fundamental. However, just a few domains have labelled data and their preparation is often unfeasible due to time constraints, economic resources required and human experts’ effort.

To attempt to resolve these problems, different self-supervised approaches have been proposed, which artificially create supervised training set from unlabeled data in order to deploy models without bottlenecks caused by high human costs in the loop for dataset labeling. A well-known self-supervised learning task is masked language modeling, where a token in a previously tokenized sentence is hidden using a mask. The model is trained to recover it, creating a probability distribution over the entire dictionary. This solution does not require labels and it is successfully used to train state-of-the-art language models such as BERT [1], RoBERTa [2], SciBERT [3], BioBERT [4] by inducing the model to discover word relationships, without knowing them a priori. Another self-supervised technique is the one proposed to train ALBERT [5]. It is called sentence-order prediction (SOP) and consists of guessing which pairs of sentences are in the correct order. SOP uses two consecutive sentences from the same documents as positive examples and as negatives the same two segments but with their order swapped. Both methods turn a large dataset of raw textual data into an informative training set proper to train powerfull neural models without human interaction. Unfortunately, these solutions are not always available for each task or domain. For this reason, finding new unsupervised approaches has become the focus of many researchers.

With this work we propose a novel self-supervised method to automatically discover semantic similarities between documents with no expert effort. Our kernel idea is to leverage the bibliography references among papers to automatically define reliable semantic relationships between them, considering that papers with common citations should have some sort of positive relationship. In other words the papers’ bibliography become valid relationships among them, like a sort of soft labelling introduced by their authors. By leveraging the bibliography references, we automatically create an informative training set of triples, where the first element of each triple is the title of a paper *q*, the second element *p* is the abstract of *q*, of course positively related to *q* itself, and the third element is the abstract of a negatively related paper *n* with *p*, namely a paper *n* that does not share bibliography references with *q*. Our approach employs this training set and deep metric learning to create a latent semantic space where the title of a paper, which is interpreted like a query by the resulting information retrieval, is placed as close as possible to its abstract and as far as possible to the negative paper with which it has been combined in the triplet. The bibliography matrix, where documents are in the rows and references in the columns, is a sparse binary matrix that summarizes the bibliography of each paper, thus the memory required to elaborate it is very limited. To further improve our results we derived indirect latent bibliography relationships among papers by applying to this sparse matrix the singular value decomposition. The improvement has been achieved because indirect relationships allowed to better identify false negative papers.

With this novel method we trained a SciBERT model with deep metric learning, namely by replacing its loss with a ranking loss in order to create the latent semantic space of corpora of papers. Then we implemented over this latent space the search engine we call SUBLIMER that we applied to the CORD19 dataset and make it available at the links https://sublimerteam.github.io/sublimer/ and https://www.unibo.it/sitoweb/gianluca.moro/useful-contents/ (accessed on 16 September 2021).

Finally, we tested SUBLIMER against the state-of-the-art (SOTA) competitors using TREC-COVID evaluation set. Our solution outperforms them in two out five most important key metrics: Precision@5 (P@5) and Bpref and achieves comparable performances in the other three measures. Differently from SOTA competitors which need supervised datasets, as CoSearch [6], SQuAD [7], HotPotQA [8], MSMarco [9], instead SUBLIMER is entirely trained on the unsupervised CORD-19 corpus with self-supervised learning with no human supervision. Furthermore, it allows a significant minor usage of memory and resources, in fact we showed that the number of trainable parameters in our whole system is an order of magnitude lower than the state-of-the-art CoSearch.

### 1.2. Organization of the Paper

The paper is organized as follows. Section 2 is about hardware and software technologies used during the project development. Then it introduces the dataset CORD-19 with related resources (i.e., TREC-COVID test set). After that, it explains our contribution deeply: the new self-supervised technique, addressing both the idea and the applications. In this section, are also provided all steps required to reproduce our work. Section 3 contains the essential experiments performed, results obtained, with an explanation of them. We validated each step of our solution using formal tests. In Section 4, we discuss our work, and we try to point out new research directions to expand our work.

### 1.3. Related Works

#### 1.3.1. Neural Language Models

For years, researchers have developed methods and algorithms to automate the extraction of valuable and structured knowledge from raw text [10,11,12,13], even with computational linguistic and algebraic approaches, such as the latent semantic analysis [14]. This research field, which is named Natural Language Processing (NLP), has produced crucial breakthroughs thanks to recent deep learning advancements. In the last years many transformers [15] based neural networks, such as BERT [1], RoBERTa [2] or XLNet [16], have led to breakthroughs in many NLP downstream tasks. For each of them there are pre-trained model, often referred as language models, which are the basis of many specific domain solutions. For instance, BioBERT [4] is specialized in the biology field, or SciBERT [3] is meant for the general scientific one and both are fine-tuned on large corpora of articles representing the state-of-the-art in their domain. The current research direction is to train larger and larger versions of this kind of models, as proved by recent publications like GPT-2 [17,18,19], GPT-3 [20], Switch-Transformer [21] and many others. Actually these works get better results, exhibit one-shot learning and some sort of reasoning capabilities. On the other hand, these models require high computational powers and training time so their further improvements are mainly achievable by big organizations endowed with large resources. SciBERT has been successfully used to create a scientific document embedder model named SPeCTER [22], which is the new state-of-the-art for different representation tasks of paper corpora. In particular SPeCTER is available with a test framework called *SCIDOC evaluation suite* that comprehends document classification, user activity prediction, citation prediction, and recommendation. This embedder model of papers represents each article with a vector of real numbers, namely a document embedding, using only its title and abstract. It employs a *triplet loss function* according to which each training instance is composed by three papers: $P^{q}$ the query paper, $P^{+}$ the paper with positive relation with the query paper, and $P^{-}$ the paper with negative relation with the query. $P^{+}$ is selected between the papers cited by the query, while the negative one is selected either among those not cited by both $P^{q},P^{+}$ (i.e., *Negative*) or among those cited just by $P^{+}$ (i.e., *Hard Negative*).

(1) $$L=max\{\left(d\left(P^{q},P^{+}\right)-d\left(P^{q},P^{-}\right)+m\right),0\}$$

In this equation, which contains the loss function used to train the neural model, *d* is the euclidean distance and *m* is a constant, called margin, that represents the minimum separation required between positive and negative related instances. The triplet loss function was firstly introduced in [23] and had a great success for the capacity to train neural models by exploiting intrinsic domain relationships. One of the first language models trained with the triplet loss is a Siamese neural model called SentenceBERT [24]. This loss has been successfully applied in different domains, such as computer vision [25,26]. Different loss functions have been proposed based on this kind of relationships, such as soft triplet loss [27], angular loss [28], multi-similarity loss [29]; a comprehensive study in [30] collects and evaluates several variants under the new research thread of *deep metric learning* and metric loss functions [31,32]. A limit of metric loss functions is that they require labelled data to define positive and negative relationships. When it is possible to automatically leverage intrinsic relationships between instances, like SPeCTER does with paper citations, we can perform a kind of training called *self-supervised learning*, where supervised algorithms are trained from unlabelled data. Following this approach the BERT model was trained with two different self-supervised classification techniques: (i) the next sentence prediction from an input sentence and (ii) the prediction of masked words in an input sentence [1]. The RoBERTa model [2] does not perform the second classification task, proving it is not necessary. ALBERT [5] is a new self-supervised solution trained by modeling inter-sentence coherence. Relationships among unlabelled data can also be artificially created in order to prepare training sets for self-supervised learning tasks. Numerous examples are also present in computer vision, like the rotation approach [33], where the model is trained to guess the angle of rotation of an image, or the Jigsaw puzzle approach [34], where a model has to guess the original position of nine pieces of a split image after a random permutation.

#### 1.3.2. Information Retrieval

The powerful language models mentioned in the previous subsection, which are the basis of several new neural information retrieval solutions, are bringing advancements in neural ranking models [35] and consequenlty in the following domains:

- *Ad-Hoc Retrieval*. It is a classical retrieval task in which a user specifies a query and the engine finds the most related documents in the corpus. The returned documents are often ranked according to the relevance to the user’s needs [36,37].
- *Question Answering*. It attempts to directly answer a user’s question, looking for the information in the textual data that could be structured (Knowledge Base) or unstructured (e.g., documents or Web pages ) [38,39,40].
- *Community Question Answering*. It looks through QA resources in Community QA websites like Quora, Stack Overflow and others to find the answer to a user’s question ([41]). Sometimes they seek similar questions and not the response because similar questions should have similar solutions [42,43].
- *Automatic Conversation*. It tries to replicate a human-like dialogue interface, for accessibility, question answering, and social chats [44,45].

Regardless of the applications, the architectures of these systems can be divided into *Symmetric* or *Asymmetric*. In the first case, the query and the documents are processed in the same way [44,46,47]. Otherwise, if there are two different ways to elaborate the query and documents, we have an Asymmetric System [48].

We can use different loss functions to train an IR system. The most straightforward idea is the *Pointwise Ranking Objective* [49] where it considers each item with its label. Another solution proposed in [49] and called *Pairwise Ranking Objectives* forces the model to gives similar scores to similar documents. A more complex solution, the *Listwise Raking Objectives*, works directly with lists of sorted documents [50].

These IR engines have been playing a central during the recent COVID-19 pandemic. Researchers have published a lot of scientific articles, creating an impressive amount of textual knowledge about this virus, collected in a dataset called CORD-19 ([51]). IR systems became fundamental to let experts, scientists, clinical extract human-readable information and find high-quality answers. One of the first created is *COVIDEX* [52] that applies state-of-the-art techniques in the IR field to this domain. They deployed it on a web app free to use at the link https://covidex.ai/ (accessed on 6 August 2021). It comprises two main stages: 1. the keyword Search done by BM25Okapi algorithm performed by Pyserini [53] framework, 2. Neural Reranker using monoT5, based on T5 [54], trained to guess if two input sequences were correlated or not. The neural model was trained with MSMarco dataset [9], a labeled dataset for information retrieval. Another example that, as far as we know, represents the state-of-the-art today is Co-Search [6]. It uses a complex architecture combined with a BERT model fine-tuned on CORD-19 Dataset. It is an ad hoc retrieval system that behaves as a Listwise Ranker on the whole corpus. It takes a textual query and produces a list of the best *N* documents, sorted according to a ranking score. Its architecture is composed by three parts: *indexing*, that turns each paragraph of a document into a vector, *retrieval*, it deals with the query and it selects the most related ones, *ranking* where the selected paragraphs are sorted according to their relevance to the user’s needs. They leveraged different neural models as SentenceBERT [24] to create semantic indices, a custom sequence-to-sequence model for summarization based on GPT2, and a model for question answering. The first was trained on CORD-19 using a self-supervised technique that exploits the relationships between paragraphs and their citations. The summarization model was also trained in a self-supervised way using abstract as target and the full-body as inputs. The QA model was trained using two different labeled datasets, the HotpotQA [8] and the PubMedQA [55]. Furthermore, they used another extractive reading comprehension model trained to SQuAD [7].

## 2. Materials and Methods

### 2.1. Hardware and Software

We realized the entire project, from the data preprocessing to the model training and web application deploying, using a workstation with a GPU Titan XP with 12 GB of dedicated memory and an Nvidia RTX 3090 with 24 GB of dedicated memory, CPU I5-6400 with four cores, and 24 GB of RAM. It uses Ubuntu 16.04.6 LTS as Operative System. For minor tasks as some tests, we leveraged Google Collaboratory. The technologies employed are python3, PyTorch framework to address Deep learning tasks, HuggingFace transformers to works with transformers models as Bert and SciBert, scikit-learn package, and nltk for data preprocessing.

### 2.2. CORD-19 Dataset

The COVID-19 Open Research Dataset (CORD-19) is a public dataset containing over 280 K scientific studies about all coronaviruses, and it is the most extensive and complete library on this topic. It is created by important tech organizations like Allen Institute for AI, Chan Zuckerberg Initiative, Microsoft, after a call to action from the White House in March 2020 (https://venturebeat.com/2020/03/12/white-house-seeks-tech-companies-ai-to-combat-coronavirus-outbreak/, accessed on 16 September 2021). It is growing week by week with new researches and papers, collected with their metadata: title, abstract, authors, publish date. Less than 45% have also the entire body in a JSON format created by using the algorithm proposed in the paper [56]. Papers do not have labels or any sort of content classification, making the use of such datasets challenging for the train of deep neural model.

Furthermore, also Text Retrieval Conference (TREC) (https://trec.nist.gov/, accessed on 16 September 2021) showed interest in the project. They created a challenge on hosted on Kaggle called *TREC-COVID Information Retrieval* where teams had to submit for each of the given queries a maximum of 1000 papers from the CORD-19 that contain the answer. They split this challenge into 5 rounds, the first contained 30 topics, and each of the following rounds added five more, but also used more recent versions of the dataset CORD19, for a total of 50 topics. They evaluated each submission using automatic tools and manual checks. In this way, they created for each round a pool of judged query-documents pairs with their relevance score. Each pair was ranked zero for non-relevant, one for partial relevant, and two for relevant. It is possible, thanks to that, to test and compare different IR engines on the CORD-19 dataset domain with solid metrics.

### 2.3. The Language Model

#### 2.3.1. Bibliography Latent Information

Train deep learning models is a complex task because it requires good knowledge of the technology, a deep understanding of the domain, and an excellent labeled training set capable to precisely define the knowledge the model has to learn. However, good training sets are often hard to find, require human supervision to be created, and are often kept private by their owners. Creating one is generally unfeasible for time constraints and economic resources [57,58]. So recent trends are born to address how to train models when data do not have labels. Principal solutions are weekly supervised learning [59,60,61] where few labeled data are present, or *self-supervised learning* [62,63]. *Self Supervised Learning* is a technique where a model learns the desired knowledge for a task as a side effect, exploiting existing relationships in the data. In this way, it is possible to create a training set in a short time and with no human supervision. Famous self-supervised tasks are masked language modelling adopted by language models to train on the specific domain as BERT [1,3] or Next Sentence Prediction used in BERT [1].

We decided to explore a new self-supervised approach for CORD19 dataset, t exploiting the bibliography relationships among papers, in order to create soft labels for the train.The principal idea is to train a neural model, with deep metric learning, to create a latent space where similar papers are close to each other while dissimilar are placed away. To achieve this, we created triplets of elements with positive and negatives relationships. Each triplet is composed by the title of a paper *q* and its abstract as positive elements, while the abstract of a dissimilar paper *n* is the negative one. In order to define such relationships, we exploited the information contained in the bibliography.

The idea we found out is that the bibliography contains semantic information about the paper itself that is possible to exploit in order to automatically create good relationships between documents, otherwise impossible to find without reading and comprehending the full documents. The literature already presents some works that sustain this claim, proposing different approaches to use it, in particular it was firstly conceptualized by the first author of this paper in [64]. Furthermore, it was used in [65] and in Specter [22] that uses direct citation to create a positive tuple. The foundation of this idea is that two given papers, where at least one cites the other, have hidden semantic relationships; they could be about the same arguments, have a joint related work, or other similarities. However, a single citation is not enough to precisely define the similarity of two documents, and it does not give us a similarity weight between them. We propose a new method that uses the entire bibliography, representing it as a vector of real number, a point in a high dimensional latent space where the position has semantic meaning. In other words, two papers mapped close to each other have more common topics than two places far away. This feature of the latent space allows the comparison between them and the definition of hidden relationships, using the spatial distance as a metric for their semantic similarity.

We studied new techniques to inject this latent information from the bibliography into the knowledge learned by a language model to make it more powerful and suitable for non-supervised learning. We crafted the training set by creating tuples of similar and dissimilar papers according to such bibliography embeddings, and then we exploited them with a triplet loss function to train a SciBERT model. In the following section, we explain step by step our approach to ensure reproducibility and transparency.

#### 2.3.2. Training Set Creation

Firstly, we created a matrix $M_{DXC}$ where *D* is the number of the documents in the CORD19 (version of 9 September 2020) and *C* is the number of all the cited papers, most of that are not present in the dataset. To reduce noise and redundancy that could create problems during training time, we dropped all cited papers with less than two references and all documents from the corpus that cite only papers not cited by any other document. In this way, we create a binary-sparse matrix with a shape of 94,037 × 422,360. The cell d,c is set to 1 if the document *d* contains *c* in its reference list, 0 otherwise. So each row summarizes, through a sparse binary vector, the bibliography of the corresponding paper. It is possible to use this to compare documents; however, the problem is that such vectors is they do not model high-order relationships. Using such structure, we can find out how many common citations two given papers have. Before go further, we need to define some key concepts: (i) exists a *First Order Relation* within $d_{0}$ and $d_{1}$ if both cite at least one common paper *c*. (ii) Between $d_{0}$ and $d_{1}$ can still exist a relationship even if they do not cite the same papers, but the cited papers are on the same arguments. We can call this relation *High Order Relation*. Climbing the citation graph to a higher level starting from $d_{0}$ and $d_{1}$, it is logical to think that we will find common papers or common citation patterns if they are semantically related. Those far elements somehow represent a relationship between $d_{0}$ and $d_{1}$, while the distance from the starting documents can represent the score to weigh it. This information is hidden inside the bibliography of documents, and we need to let it emerge.

This problem is very similar to the latent semantic space construction problem, well known in NLP, where they leverage the frequency of words in a text to extract semantic meaning. We can apply this solution in our case if we consider the bibliography as our text and citations as our words.

To solve the problem of the hidden latent information, we used a well-known solution in NLP for the creation of the latent semantic space: the singular value decomposition (SVD). It reduces the dimensionality of the matrix and makes hidden relationships appear. It was successfully used to model text-based domains by GM in his recent works [66,67,68]. We empirically set *k* equals 1024. The latent space created in this way L was used to find similar papers, comparing the resulting vector of the paper bibliography through the Cosine Distance. In this way, we can now place a document into a latent space, and automatically study its relationships with others without reading the full text.

**Figure 1:**
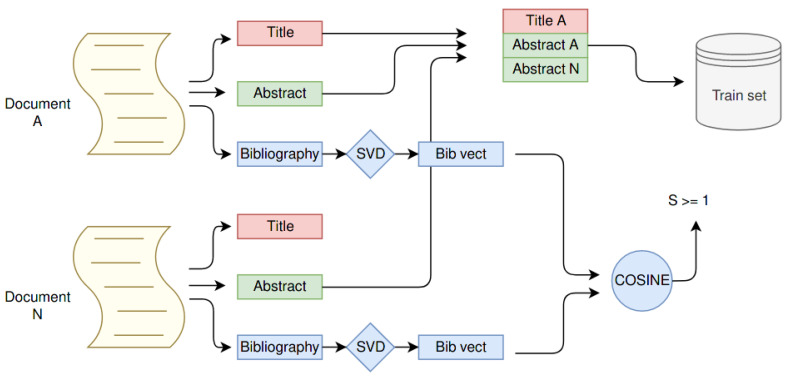
The picture shows how the training set is created. It turns the bibliography of two papers into vectors and then checks their cosine distance. If it is higher or equal to 1, it uses the N document as a negative one for paper A. It creates the training sample by using the title and the abstract from A and only the abstract from N.

Using this tool, we created a training set for the neural model learning coupling two elements: the query paper $P_{q}$ and the negative $P_{n}$. First, we pick all the elements with full-body available and the title not null, for a total of 90K documents. Then for each of them, we select a real negative by using the bibliography structure, selecting as $P_{n}^{\prime }$ only papers which their bibliography vector has a cosine distance to $P_{q}$ greater than 1. For each $P_{q}$, we selected three $P_{n}$ creating three training instances. In this way, the training set reached 270K different samples. Each sample was composed of the title and the abstract from $P_{q}$ and $P_{n}$. We show this process graphically in Figure 1.

#### 2.3.3. Loss Function

We found out that the best way to train a model on this dataset and exploiting relationships was to use a *triplet loss function* as an objective function for the training. Other successful neural models used it as SPECTER [22]. We defined the loss function as:

(2) L=max(dp−dn+m,0)

This function takes 3 elements $e_{q}^{t},e_{q}^{a}$ which are the embeddings of the title and the abstract of the query paper, and $e_{n}^{a}$ the embedding of the abstract from the negative paper. We defined dp as the Euclidean distance between $e_{q}^{t}$ and $e_{q}^{a}$, dn as the Euclidean distance between $e_{q}^{t}$ and $e_{n}^{a}$. *m* represents the margin that means how close informative negatives have to be.

(3) $$d\left(P,P^{\prime }\right)=\sum_{i=0}^{i=|P|}{\left(P_{i}-P_{i}^{\prime }\right)}^{2}{]}^{\frac{1}{2}}$$

**Figure 2:**
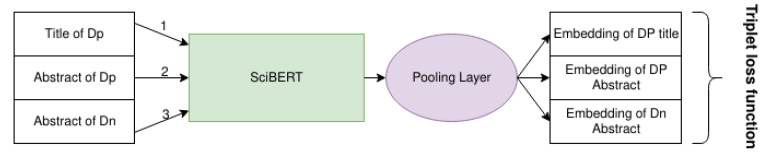
In the picture we show how SciBERT model is trained. Numbers on the input items indicate that they are process sequentially.

The idea is to train the model to generate embeddings and put them closer if they are parts of the same paper than the title and the abstract of two different ones. In this way, the model correctly links a brief sentence as the title to a long one as its abstract. This method is helpful to answer user queries, which are shorter than a document, and find more hidden semantic associations between the papers. We provide a graphical description of the inputs and outputs of SciBERT during training in Figure 2.

We also tried to train our model using multi similarity loss [29], state-of-the-art in deep metric learning. It requires a pool of positive examples and a pool of negatives. We used a miner capable of find out hard negative relationships and different similar relationships. We tried different combinations, but the best we find out is to use the title, abstract, introduction, and conclusion of a paper as positive and abstract, introduction, and conclusion of a negative paper as negative elements. We also tried with more positive items using three similar papers and ten different abstracts from negatives papers as negative items, but we did not improve the results.

We started from a pre-trained language model. In particular, we selected SciBERT [3] because it already contains knowledge on the scientific articles’ domain, and we did not perform any structural change to the architecture of the network. One of the crucial advantages of using a pre-trained network is that they require less time to be fine-tuned but still they reach the best results. However, we needed a model capable of generating sentence embeddings, so we trained it with the triplet loss function, forcing it to generate comparable and meaningful vectors. SciBERT uses a specific token dictionary of 31,090 elements and, before giving some text to it, it has to be converted into tokens. The model produces a vector of 768 dimensions representing a point in the latent space for each of these tokens.

(4) $$Y^{S}=M\left(I_{IDs}^{S}\right)$$

In the formula, we can see the input $I_{IDs}^{S}$ that represents the array of 512 tokens from the sentence *S*. The model *M* reads it and produces $Y^{S}$ that is a matrix 512 × 768. Then, to create Sentence Embedding, we decided to combine all output embeddings in one by using the mean.

(5) $$E^{S}=mean\left(Y^{S}\right)$$

In this way, it produces $E^{S}$ that is a vector of 768 dimensions representing the input sentence.

### 2.4. The IR System

**Figure 3:**
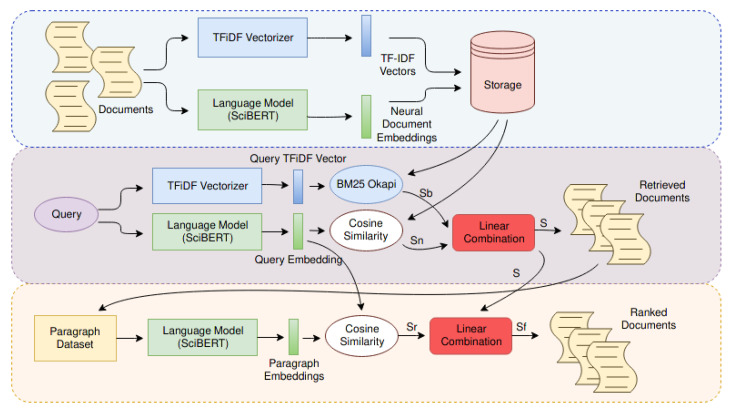
The picture shows the entire system from the dataset creation to the ranked documents. Inputs of the system are the documents to create data structures and queries. The system uses the same elements to treat documents or queries, and then it looks for documents related to the query using Neural Embeddings and the BM25 Okapi algorithm. After that, it sorts the retrieved papers using paragraph information. The ranked documents are the output of the system.

We placed this new language model in a complete ad-hoc information retrieval system called SUBLIMER. It was built on the top of the CORD-19 dataset, but it can be deployed on each scientific domain because it does not need any labels to work. The entire architecture is shown in Figure 3, and it can be divided into three modules:

- *The indexer*. It makes use of two embedding techniques to create different data structures useful for indexing: a real number vector representing the whole document (i.e., Neural Embedding), created by the language model, and the term frequency vector (i.e., TF-iDF) alongside with the bag of words of the entire domain used by the BM25 Okapi search algorithm [69].
- *The retriever*. It takes a query expressed by natural language, turns it into a vector using the neural network, and computes the TF-iDF. Then, the neural embedding is used to find the semantic related documents through cosine similarity, while BM25 Okapi algorithm leverages the tf-vector to assign to each document a score. Results from both the techniques are combined, and then all documents are sorted according to this new score.
- *The reranker*. The main idea behind this module is the title and the abstract used until this step to represent the entire document are not enough because some information remains unveiled in the full body. For this reason, this module considers all the inner paragraphs that compose the first K documents, in order to sort them according to their content. This task is performed by using a Neural Ranker Model, which is the same used by Retriever.

#### 2.4.1. The Indexer

This module is meant to create indices and data structures useful to represent each document’s syntax and semantic, and easily fetch documents related to a given query. It is fundamental to approach this phase considering both the semantic of a document and its syntax structure, combining this information. We decided to represent the first by using the neural model SciBERT fine-tuned on CORD19 using our novel self-supervised approach explained in Section 2.3. It takes as input the title $t_{d}$ and the abstract $a_{d}$ of a document d∈D and produces a neural embedding $e_{d}$ of 768 dimensions. For the second aspect, we decided to use a keyword algorithm, in particular the BM25 Okapi [69] that is considered the best tf-based algorithm. We created a dataset of titles and abstracts of each document, and we tokenized them. After that, we created a bag of words, and for each word, the inverse document frequency (iDF).

(6) $$iDF\left(w_{i}\right)=\ln\frac{N-n(w_{i})+0.5}{n(w_{i})+0.5}+1$$

where *N* is the number of documents in the corpus D, $n(w_{i})$ the number of documents d∈D containing $w_{i}$. Then, for each document *d*, we created the term frequency vector $tf_{d}$. At this point, all documents are represented by a tf-vector and a neural embedding.

#### 2.4.2. The Retriever

This module processes the query and returns all documents sorted by their meaning to the given topic exploiting the data structures created by the indexer. Firstly, the query is given to the neural model that generates the embedding $e_{q}$, and after that, the system constructs the tf-idf vector $v_{q}$. The neural representation $e_{q}$ is then compared to all documents embeddings $e_{d}\forall d\in D$ creating a neural score $s_{n}$ based on the cosine similarity.

(7) $$s_{n}\left(q,d\right)=CosSim\left(e_{q},e_{d}\right)$$

(8) $$CosSim\left(a,b\right)=\frac{\sum_{i=1}^{d}a_{i}b_{i}}{\sqrt{\sum_{i=1}^{d}a_{i}^{2}}\sqrt{\sum_{i=1}^{d}b_{i}^{2}}}$$

where d is the dimension of the vectors. Alongside the neural score, we used BM25 Okapi to compute a second one $s_{b}$ for each document:

(9) $$sb\left(q,d\right)=\sum_{i=0}^{\left|q\right|}IDF\left(q_{i}\right)\cdot \frac{f\left(q_{i},d\right)\cdot \left(k+1\right)}{f\left(q_{i},d\right)+k\cdot \left(1-b+b\cdot \frac{\left|d\right|}{avgdl}\right)}$$

where IDF(w) returns the inverted document frequency of the word *w*, f(w,d) returns the frequency of the word *w* in the document *d*. avgdl is the average length of documents in the dataset. Then, we assigned to each document one score *s* as the result of a linear combination of both $s_{n}$ and $s_{b}$.

(10) s=α∗sn+(1−α)∗sb

with α that is a real number between 0 and 1. At the end of this phase, the entire dataset is sorted according to this final score that weight the relationships between the document and the given query.

#### 2.4.3. The Ranker

At this point, we have all documents $d_{0}^{\prime },d_{1}^{\prime },\cdots ,d_{n}^{\prime }$ sorted according to their relevance to the input query. The system created this rank considering only the title and abstract without any insight into the entire body’s information, so we decided to address this problem by adding a ranker model to the top of the IR pipeline. In order to keep the whole system as light as possible, we decided to reuse the neural model from previous phases, but applying it paragraphs level. In this way, our solution can enrich the quality of the selected papers with full text information of each one and improve their order from retrieval step. However, it was impossible to process the entire dataset in a reasonable time, so we defined a new hyperparameter called *Pool* *p* that represents the number of papers to use in this phase from the top of the output list of the retrieved documents. The system now creates a new temporary dataset composed of all paragraphs from such pool. However, some of them could lack the full text, in order to avoid empty entry, we added also the title and the abstract of each paper to the paragraphs’ dataset. In this way, there are at least two entries for each document. Similar to the retrieval phase, the system turns each instance of the dataset into an embedding. For each document, it takes the max score from the most similar paragraph. Then it computes the score $s^{r}$, by using cosine similarity with the user query.

(11) $$sr_{j}={\mathrm{argmax}}_{p_{j}}(\mathrm{Similarity}\left(R\left(p_{j}\right),e_{q}\right)$$

where $sr_{j}$ is the score from the ranker for the document *j*, $p_{j}$ represents all paragraphs plus the title of $d_{j}$, R(x) is the embedding created by the model for the input *x* and $e_{q}$ is the query embedding directly from the retriever. At this point, each document has two scores *s* from the retriever and sr from the reranker. We decided to create a unique score sf combining them:

(12) sf=β∗s+(1−β)∗sr

with β between 0 and 1. In this way, the IR system can find papers that contain valuable information also in the body and not only in the abstract.

#### 2.4.4. IR System Configuration

First, we trained the model starting from SciBERT for three epochs. We used *Adam* optimizer with a learning rate of $5e^{-6}$ and a batch size of 1 on a GPU Nvidia Titan XP with 12 GB of graphic memory. Then we created a BM25 Okapi model with K = 1.25 and B = 0.75. Each sentence, before BM25 Okapi, was tokenized using an English word tokenizer from the NLTK framework. Then we found out that the best α was 0.815, the best Pool *p* was 10, and the optimal β was 0.77. When we speak of SUBLIMER, we refer to this configuration.

### 2.5. Language Model Fine-Tuning with Teacher

As expected, once the entire IR engine was ready and tested, we found out that the neural model alone reached worse results than the entire system. That is pretty obvious, but it is the foundation of the next step. We tried to improve the neural network only using a new training set created by the entire IR. The latter can judge a document using more information, because it checks the body and combines semantic and syntactic analysis. We wanted to enrich the language model knowledge with this extra information, so we created a new training set to fine-tune it by taking the output from the entire system. Let us define $y_{q}=<d_{0},\cdots ,d_{n}>$ the set of documents retrieved by the whole IR for the query *q*:

(13) $$y_{q}=<d_{0},\cdots ,d_{n}>=IR\left(q\right)$$

We selected the first three papers $d_{0},d_{1},d_{2}$ as the set of the positives. Then we selected the last 15 papers as the negatives. For each positive we assigned five negatives sequentially, in this way $d_{0}$ was paired with $d_{n},d_{n-1},\ldots ,d_{n-4}$, $d_{1}$ with $d_{n-5},\ldots ,d_{n-9}$, and so on. The queries used are the topics from round 1, and the base dataset came from the test set. In this way, we created a new training set of 450 instances composed of the title and the abstract of the positive documents and the abstract from the negative one. We fine-tuned our language model for two epochs using a learning rate of $5e^{-6}$ and Adam optimizer and the triplet loss function. We refer to the full IR system based on this new model as SUBLIMERft. It got even better results than the base IR. Furthermore, we did not use any labels to performs this second fine-tuning.

## 3. Results

We performed a series of tests to formally evaluate the entire system and its components as the language model. The goal was to analyze the different configurations of SUBLIMER against Co-Search and COVIDEX, state-of-the-art on CORD19 information retrieval. We put all our efforts into creating fair comparison between these systems. Competitor were fine-tuned using different labeled datasets, while SUBLIMER was trained only on CORD-19. In the scope of our study, this is a difference we want to highlight to show the differences between models trained using different supervised learnings and our solution that uses only a training set created automatically starting from the not labeled CORD-19 dataset. Moreover, competitors also used CORD-19 to train part of their models as described in Section 1.3.2. Furthermore, we removed all common papers between training set and test set, as we discussed in Section 3.3. For these reasons, the comparison is fair and sound. For the formal evaluation, we used the TREC-COVID Test set and standard retrieval metrics for the evaluation.

### 3.1. TREC-COVID Test Set

In response to the COVID-19 pandemic, the Text Retrieval Conference (TREC), with the collaboration of the National Institute of Standards and Technology (NIST), created an evaluation dataset for coronavirus IR systems [70] as we explained in Section 2.2.

They created a labeled dataset for each round to test the IR systems. Each topic is expressed with three different levels of verbosity: topic name, a human-formulated question, and a narrative. The performances of the tested systems are evaluated by different standard metrics, including P@5, P@10, nDCG@10, MAP, and Bpref. Each document can appear in more topics, with a different score for each of them.

### 3.2. Evaluation Metrics

TREC defines a series of metrics to evaluate different aspects of IR systems quantitatively: precision at a different level (P@5, P@10), nDCG that considers the position of the retrieved documents, MAP, and Bpref that works fine in situations of missing relevance judgments.

#### 3.2.1. Precision

It checks the number of relevant documents within the retrieved ones:

(14) $$P@N=\frac{\left|\mathrm{relevant}\mathrm{documents}\mathrm{in}\mathrm{top}-N\right|}{N}$$

#### 3.2.2. nDCG

Normalized discounted cumulative gain performs:

(15) $$nDCG@N=\frac{1}{Q}\sum_{q=0}^{Q}\frac{DCG^{q}}{IDCG^{q}}$$

where *Q* is the number of queries, $DCG^{q}$ is the discounted cumulative gain of the query *q* and it is computed as:

(16) $$DCG^{q}=rel_{1}^{q}+\sum_{i=2}^{N}\frac{rel_{i}^{q}}{log_{2}\left(i\right)}$$

$rel_{i}^{q}$ is the relevance of the retrieved document in the position *i* with respect to the topic *q*. The $IDCG^{q}$ is the ideal DCG or the highest possible DCG. nDCG performs reliably in measuring search engine performance.

#### 3.2.3. MAP

Mean average precision is the average precision of the retrieved document set, and it is defined as the integral over the normalized precision-recall curves of the query set. It is defined as:

(17) $$MAP=\frac{1}{Q}\sum_{q=1}^{Q}\int_{0}^{1}P_{q}\left(R\right)dR$$

where *R* is the recall, $P_{q}$ is the precision expressed as a function of the recall for the query *q*.

#### 3.2.4. Bpref

Binary preference [71] uses information from judged documents, and it is very robust in the context of incomplete relevance judges. It checks how frequently irrelevant documents are retrieved before relevant. It is formulated as:

(18) $$Bpref=\frac{1}{R}\sum_{r=1}^{R}1-\frac{\left|n\mathrm{ranked}\mathrm{higher}\mathrm{than}r\right|}{R}$$

where *R* is the number of judged relevant documents, *r* is a relevant retrieved document, *n* is the number of irrelevant retrieved documents.

### 3.3. IR Results

We tested our solution against the first round of the TREC-COVID test set, comparing our results with the state-of-the-art in this domain: CO-Search and COVIDEX. The first round is composed of 8690 judged pairs paper-topic, for a total of 4778 papers. Each pair is evaluated with a score of 0, 1, or 2 according to the relation with the topic. For each topic, we selected the best 1000 retrieved documents, and we evaluated them using the tool pytrec_eval.py https://github.com/cvangysel/pytrec_eval (accessed on 16 September 2021) that is a python wrapper of the trec_eval https://github.com/usnistgov/trec_eval tool created by TREC (accessed on 16 September 2021). In Table 1, we compared the same model but trained with different methods. We considered the model alone and the whole information retrieval structure. First, we present results obtained using the triplet loss function but without using bibliography information. In this case, the negative paper was selected randomly within the whole dataset. Then we show the results of both models trained with triplet loss and multi similarity loss but selecting the negative paper leveraging the bibliography embeddings. They performed better than the first trained without considering the bibliography, confirming our idea to exploit this hidden relationships. Moreover, the second two got similar performances, but the model trained with the triplet loss function performs slightly better on precision, so we used this model for the comparison with state-of-the-art.

We show in Table 2, our solution reached the competitors CoSearch and COVIDEX, getting better results in two evaluation metrics (Precision@5, and Bpref), but leveraging only a self-supervised learning. However, it does not completely overcome the state-of-the-art, and we have no interest in that. We just want to prove the quality and the power of our solution that allowed us to get state-of-the-art performances but using no labels and with significantly fewer parameters (see Section 3.4) than competitors. Furthermore, we repeated the experiment using the same training set but removing all papers from the evaluation set. They are two completely different tasks, and joint papers do not influence the validity of the results; in fact, competitors do not perform this kind of test. As expected, we did not appreciate differences in the results, infact the test task is about finding related papers for a given query within a pool of documents, while the training task is to improve the positioning of the documents in the latent space.

Finally, in Table 3, we present how single components of our information retrieval system contribute to the final score. We gradually increase α to show the contribution of BM25 Okapi to the retrieved phase. We avoid the use of the reranker in this first analysis. Then we set α to 0.815, and we add it. Moving β, we showed how it improves the final results.

### 3.4. System Size Comparison

We also want to show that our entire informational retrieval system has significantly fewer trainable parameters than CoSearch [6], the current state-of-the-art. The neural model adopted in our work is SciBERT that uses the same architecture of BERT and according to [72] BERT counts 110 M parameters. We use the same model two times, whitout fine-tuning it, one for the retrieval and one for the reranker, with BM25 Okapi that does not have trainable parameters. So we can affirm that our whole system has a total of 110 M parameters. CoSearch is built using different neural models: SciBERT (110 M parameters), a summarization model composed by BERT (110 M) as encoder, and a modified GPT-2 (1.5 B). Then they also use a question-answer model without specifying the architecture, so we cannot estimate the number of parameters (X). Their whole system has a total 1720 B + X parameters, but the authors did not release architecture details, so we computed them using the available information from their paper. According to them, our system has a number of parameters an order smaller than state-of-the-art CoSearch. This is a fundamental ingredient to let small researcher groups work with information retrieval systems because fewer parameters to train means cheaper hardware required and less time.

### 3.5. Bibliography Embeddings Evaluation

We performed a test by using labeled documents from TREC-COVID to formally evaluate the quality of the bibliography embeddings created by applying SVD to the bibliography matrix, as we explained in Section 2.3.2. We expected that good embeddings’ representation would place closer papers of the same topics. In particular, papers relevant to a given topic should have bibliography embeddings closer to each other than random papers. The same consideration can be done for papers with relevance two respect a given topic, and papers with relevance one to the same topic. We tested such embeddings by selecting all labeled documents from TREC-COVID with bibliography information. Then we selected 1000 random pairs from that pool as the baseline. After that, we selected 2500 pairs of papers with relevance two to the same topic (we called it R2) and 2500 pairs with relevance one (called R1). Then we create the bibliography embedding for each element, and we computed the cosine distance for each pair. Results proved that our supposition was correct. In Table 4, we show them using different K. For each K, the average distance of the baseline was higher than R1 and R2 average distances. R2 always got the lowest average distance.

### 3.6. SUBLIMER: Web Information Retrieval Application

Finally we released a full functional version of SUBLIMER as a web information retrieval applied to the COVID literature https://sublimerteam.github.io/sublimer/ (accessed on 16 September 2021). The main web page offers to the user the possibility to submit an arbitrary query, to modify the information retrieval hyperparameters as α,β, which regulate respectively the BM25Okapi and the reranker contributions to the final ranked list, and the number of documents to be used for the ranker phase. There is a dedicated section to perform the TREC-COVID test, where the user can choose one of the predefined questions and check the quality of the response through the metrics displayed in the corner. Furthermore, we added an extra feature based on the language model itself. Users can open each retrieved document in a separate window, and the system automatically performs a semantic search inside the whole article, highlighting the most relevant parts. In particular, it selects all those textual sentences within a cosine distance threshold to the query. In this way, the user can test the IR’s functionality and the language model consistently and practically. We also proved that our solution is mature enough to be deployed for a real case scenario.

## 4. Conclusions

We proposed a new self-supervised method to create a latent semantic space from unlabelled corpora of papers, where the spatial proximity among them represents their semantic similarity. However in unsupervised corpora of papers, such as the CORD-19 that contains a large collection of the COVID literature, is unknown which papers are positively and negatively related each other.

To create such a latent space the method creates a training set composed by triplets of elements: the title and the abstract of each paper *q*, which are two elements positively related, and the abstract of a dissimilar paper *n*, which is negatively related with *q* according to an unsupervised criterion. The core idea of our self-supervised method is to exploit the bibliography references among papers to define which are positively or negatively related each other. In particular the criterion to consider negatively related two papers is that they do not share bibliography references. Then, using deep metric learning, we automatically exploited these relationships to train a language model on the unsupervised CORD-19 literature, in self-supervised manner, in order to create a latent semantic space over which we implemented SUBLIMER, an efficient metric information retrieval.

We proved that our method outperforms CoSearch, the state-of-the-art on TREC-COVID, in two evaluation metrics, precision@5 and Bpref, using significantly fewer trainable parameters. This proved that (i) our self-supervised approach can compete with supervised learning without depending on previously labeled data; that means no human efforts and automatic training set creation. (ii) the bibliography references contain valuable knowledge that can be extracted and used to create solid relationships between documents and fill the labels’ lack.

The literature shows that bigger language models perform better than basic ones and we could use them to beat competitors with higher margin. However, we preferred to focus our efforts on providing a SOTA self-supervised solution for low-resources regimes, also to supply a contribution in the direction of the democratization of the modern artificial intelligence.

Future researches should investigate the best number of the low rank dimension of SVD to let emerge the indirect bibliography relationships among papers in order to reduce false negative relationships among papers. Our method is general both from the application perspective, in fact it is applicable to all unlabeled paper-based datasets, and from the technological view point by using new more powerful ranking losses and language models.

## Figures and Tables

**Table 1:** The table compares results from the model trained with triplet loss function *tml* and the one trained using the multi similarity loss msl. We also add the model’s results trained using the triplet loss function, but using random negatives instead of one selected by using bibliography (rnbl). We compare the language model *LM* but also the entire informational retrieval *SUBLIMER*.

	P@5	P@10	nDCG@10	MAP	Bpref
**LMrnbl**	0.0.69333	0.5868	0.6191	0.2716	0.5147
**SUBLIMERrnbl**	0.7867	0.69	0.6696	0.3272	0.5411
**LMtml**	0.74667	0.65333	0.6221	0.2776	0.5129
**SUBLIMERtml**	**0.8333**	**0.71**	0.6647	0.3238	0.5306
**LMmsl**	0.7000	0.64	0.6596	0.3040	0.5428
**SUBLIMERmsl**	0.8067	0.7067	**0.7109**	**0.377**	**0.5662**

**Table 2:** The table shows the results of state-of-the-art solutions on the trec-covid test set with sublimer results. We can see that our solution outperforms competitors on P@5 and Bpref.

	P@5	P@10	nDCG@10	MAP	Bpref
**CoSearch**	0.8267	**0.7933**	**0.7233**	**0.4870**	0.5176
**Covidex**	0.6467		0.6032	0.2601	
**LM**	0.74667	0.65333	0.6221	0.2776	0.5129
**SUBLIMER**	0.8333	0.71	0.6647	0.3238	0.5306
**SUBLIMERft**	**0.84**	0.7267	0.688501	0.362171	**0.556162**

**Table 3:** The table shows the contribution of BM25 Okapi regulated by *alpha* and the reranker regulated by *beta*. In the first part of the table, results refer to SUBLIMER without reranker only neural model and BM25 Okapi, while in the second part, we analyze the reranker using the best alpha 0.815.

Alpha	P@5	P@10	nDCG@10	MAP	Bpref
**1**	0.74667	0.65333	0.6221	0.2776	0.5129
**0.9**	0.74667	0.69	0.64426	0.305501	0.52505
**0.815**	**0.8067**	**0.71**	0.6633	0.3232	**0.5305**
**0.8**	0.80	0.7067	0.6656	**0.3251**	**0.5305**
**0.7**	0.76	0.6933	**0.6669**	0.328096	0.5232
**0.6**	0.7333	0.6633	0.65996	0.3183	0.5041
**Beta**	**P@5**	**P@10**	**nDCG@10**	**MAP**	**bpref**
**0.8**	0.8133	**0.71**	0.6638	0.3233	**0.5306**
**0.77**	**0.8333**	**0.71**	0.66471	**0.323768**	**0.5306**
**0.7**	0.7933	0.7067	**0.6658**	0.3233	**0.5306**

**Table 4:** This table summarizes the bibliography evaluation performed by calculating the average distance between random papers (Baseline) papers of relevance 2 (R2) or 1 (R1) to the same topic.

K	Baseline	R1	R2
2048	0.992	0.958	0.939
1024	0.991	0.943	0.922
512	0.989	0.939	0.913
256	0.981	0.906	0.871

## Data Availability

Dataset used for the train of the model is public and available at https://www.semanticscholar.org/cord19/download (accessed on 16 September 2021). All steps to recreate the training set are reported in the paper, however we can provide it upon request. Test set is also public and available at https://ir.nist.gov/covidSubmit/data.html (accessed on 16 September 2021).

## Abbreviations

The following abbreviations are used in this manuscript:

AI	Artificial Intelligence
DL	Deep Learning
IR	Informational Retriever
NLP	Natural Language Processing
LM	Language Model
TF	Term Frequency
iDF	Inverse Document Frequency
TLF	Triplet Loss Function
MSL	Multi Similarity Loss
G.M.	Gianluca Moro
L.V.	Lorenzo Valgimigli
